# Accurate diagnosis and treatment of sacral meningeal cysts without spinal nerve root fibres: identifying leakage orificium using high-resolution spherical arbitrary-dimensional reconstructing magnetic resonance imaging

**DOI:** 10.3389/fneur.2024.1298477

**Published:** 2024-01-31

**Authors:** Chenlong Yang, Xiaohui Lou, Lina Huang, Qianquan Ma, Xiaoliang Yin, Qiang Zhao, Chao Wu, Haibo Wu, Jianjun Sun

**Affiliations:** ^1^Department of Neurosurgery, Peking University Third Hospital, Peking University, Beijing, China; ^2^Department of Neurosurgery, The Third Affiliated Hospital of Wenzhou Medical University, Wenzhou, China; ^3^Department of Pain, The Third Affiliated Hospital of Wenzhou Medical University, Wenzhou, China; ^4^Department of Radiology, The Third Affiliated Hospital of Wenzhou Medical University, Wenzhou, China; ^5^Department of Radiology, Peking University Third Hospital, Peking University, Beijing, China; ^6^Department of Neurosurgery, Beijing Friendship Hospital, Capital Medical University, Beijing, China

**Keywords:** sacral cyst, spinal nerve root, magnetic resonance imaging, sectional reconstruction, leakage orificium

## Abstract

**Objective:**

This study aimed to develop an arbitrary-dimensional nerve root reconstruction magnetic resonance imaging (ANRR-MRI) technique for identifying the leakage orificium of sacral meningeal cysts (SMCs) without spinal nerve root fibres (SNRFs).

**Methods:**

This prospective study enrolled 40 consecutive patients with SMCs without SNRFs between March 2021 and March 2022. Magnetic resonance neural reconstruction sequences were performed for preoperative evaluation. The cyst and the cyst-dura intersection planes were initially identified based on the original thin-slice axial T2-weighted images. Sagittal and coronal images were then reconstructed by setting each intersecting plane as the centre. Then, three-dimensional reconstruction was performed, focusing on the suspected leakage point of the cyst. Based on the identified leakage location and size of the SMC, individual surgical plans were formulated.

**Results:**

This cohort included 30 females and 10 males, with an average age of 42.6 ± 12.2 years (range, 17–66 years). The leakage orificium was located at the rostral pole of the cyst in 23 patients, at the body region of the cyst in 12 patients, and at the caudal pole in 5 patients. The maximum diameter of the cysts ranged from 2 cm to 11 cm (average, 5.2 ± 1.9 cm). The leakage orificium was clearly identified in all patients and was ligated microscopically through a 4 cm minimally invasive incision. Postoperative imaging showed that the cysts had disappeared.

**Conclusion:**

ANRR-MRI is an accurate and efficient approach for identifying leakage orificium, facilitating the precise diagnosis and surgical treatment of SMCs without SNRFs.

## Introduction

Sacral meningeal cysts (SMCs) are cerebrospinal fluid (CSF)-filled extradural meningeal cysts that are most frequently found within the sacral canal and have a prevalence of 4.6%–13.8% in the adult population (1). SMCs are usually asymptomatic, and conditions are usually encountered during radiological screening for sensory discomfort, such as lumbosacral and lower extremity pain and numbness, in neurosurgical, orthopaedic, pain medicine, sports medicine, neurological, and other outpatient clinics (2–4). Patients are also commonly diagnosed when they are examined for anal abnormal sensations, perineal discomfort, or sexual dysfunctions in urology or gynaecology departments (5–7).

In approximately 10%–20% of patients, SMCs may cause symptoms, including local pain, numbness, soreness, and swelling in the lumbar, hip, leg, and perineal areas after prolonged walking or standing, remarkably impairing an individual’s quality of life (8–10). In severe cases, patients may experience sexual dysfunction, urinary frequency, constipation, and even urinary and faecal incontinence (2, 3). Therefore, timely and effective surgical treatment is necessary to avoid irreversible nerve damage (7).

Clinically, SMCs can be classified into two types: one type contains spinal nerve root fibres (SNRFs) within the cyst, and the other type lacks SNRFs (2). Surgical strategies vary considerably for these two variants (7). For SMCs with SNRFs, reconstruction of nerve root sleeves can prevent disease progression (2), while for SMCs without SNRFs, the surgical goal should be cyst neck ligation. Notably, SMCs without SNRFs can be further divided into several subtypes according to the pathogenic mechanism involved, including arachnoid hernia, fistula, and filum terminal leakage (2). Currently, identification of the leakage orificium is challenging, which poses great difficulties for surgeons in designing the incision and surgical extent (3). For accurate diagnosis and treatment via minimally invasive procedures, preoperative assessment of the location, size, and leakage orificium of the cysts is necessary (11).

In this study, we used specific thin-slice magnetic resonance images with high resolution and minimum layer thickness; additionally, arbitrary-dimensional reconstruction and layer spacing technology were applied to obtain the optimum images. Using arbitrary-dimensional nerve root reconstruction magnetic resonance imaging (ANRR-MRI), we identified the leakage orificium of SMCs without SNRFs, and its value for accurate diagnosis and treatment was demonstrated.

## Materials and methods

### Patients

This prospective study enrolled 40 consecutive patients who were diagnosed with SMCs without SNRFs between March 2021 and March 2022. All these patients had symptoms and signs, providing a definitive indication for surgical treatment. All SMCs containing any identifiable SNRFs within the cyst on neuroimaging were excluded. This prospective study was approved by the Institutional Review Board and Ethics Committee of Peking University Third Hospital (M2021048).

### Radiological processing

Neuro-microstructure reconstruction was performed based on MR 3D fast imaging employing the steady-state acquisition (3D-FIESTA) technique, an improved version of the 3D FIESTA sequence, using a Discovery MR750 3.0T MR system (GE Healthcare, Milwaukee, WI, United States) with a spinal coil. The scans included conventional T1-weighted, T2-weighted, and three-dimensional T2-weighted (3D-T2WI) axial nerve root reconstruction sequences. The 3D-T2W axial original images were then transferred into a GE AW4.6 reformat and 3D-MIP workstation.

High-resolution arbitrary-dimensional spherical curve planar reformation was performed. Based on the 3D-T2W axial thin-slice images, the cyst and the cyst-dura intersection planes were initially identified. Sagittal and coronal images were then reconstructed by setting each intersecting plane as the centre. Then, three-dimensional reconstruction was performed, focusing on the suspected leakage orificium of the cyst. The reconstructed images were subsequently imported into the PACS imaging system, after which the location and size of the SMCs were assessed.

### Surgical plan and intraoperative monitoring

On the high-resolution 3D-FIESTA-C images, the cyst and SNRFs were clearly displayed in two-dimensional form. Based on the reconstructed images, a surgical plan was designed that included the exact location of the cyst leakage orificium, the skin incision, and the bone window.

The locations of the cyst leakage orificium were classified into upper, middle, and lower segments, and the precise incision was made by setting the leakage orificium as the centre. Leakage was further classified into four types: arachnoid hernia, fistula, filum terminal leakage, and spinal dura mater leakage. Additionally, the maximum diameter of the cyst was measured as a preliminary preparation for designing a precise surgical incision and formulating a surgical plan.

Intraoperatively, electrophysiological monitoring was used to guarantee neural integrity, especially when the nerve structures were dissected from the cyst wall. Under the microscope, the cyst was opened to determine whether the nerve structures penetrated through or adhered to the wall internally. For larger cysts, after the leakage was sutured or ligated, the upper- or lower-pole distal cyst residue was left untreated.

### Postoperative follow-up

Postoperatively, patients were followed up at specific intervals (1 week postoperatively, 3 months postoperatively, 6 months postoperatively, and 1 year thereafter) to monitor the recovery of neurological dysfunctions, residual symptoms, and radiological absorption of the untreated portion of the cyst.

## Results

### Clinical and radiological characteristics

This cohort included 30 (75.0%) females and 10 (25.0%) males. The age of the patients ranged from 17 to 66 years, with a mean of 42.6 ± 12.17 years. The leakage orificium was located at the rostral pole of the cyst in 23 (57.5%) patients, at the body region of the cyst in 12 (30.0%) patients, and at the caudal pole (12.5%) in 5 patients. The maximum diameter of the cysts ranged from 2 cm to 11 cm, with a mean of 5.2 ± 1.9 cm.

**Figure 1:**
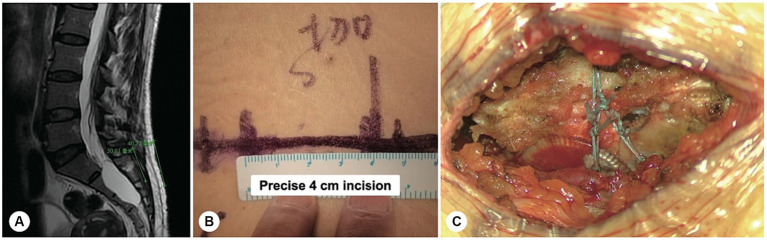
A precise incision was made for a sacral meningeal cyst without spinal nerve root fibres. **(A)** Spinal T2-weighted MR image demonstrating a sacral meningeal cyst without spinal nerve root fibres inside, and a precise incision was made as indicated by the green line. **(B)** The precise incision on the skin was only 4 cm in length. **(C)** After the cyst neck was ligated, the posterior wall of the sacral canal was reset using bone fixators.

With the use of nerve root magnetic resonance reconstruction technology, the cystic leakage orificium was accurately identified in all patients preoperatively, with an accuracy of 100%. Based on the magnetic resonance images, a precise minimally invasive incision was designed with a length of 4–5 cm, and intraoperatively, the skin incision was made based on the bone landmarks, including the crista iliaca and the coccygeal tip. The duration of the operation ranged from 1.5 to 2.5 h, with a blood loss of only 10–30 mL (Figure 1).

Among these patients, 38 had an isolated cyst, two had multiple cysts, and two had concomitant sacroanterior cysts. Based on the leakage morphology, 15 (37.5%) patients had filum terminale cysts, 14 (35.0%) had an arachnoid hernia, 6 (15.0%) had spinal dura mater leakage, and 5 (12.5%) had fistulas. Among all the cysts, 34 (83.0%) were located in the midline, 4 (9.7%) were on the left side, and 3 (7.3%) were on the middle line.

### Neurological assessments

**Figure 2:**
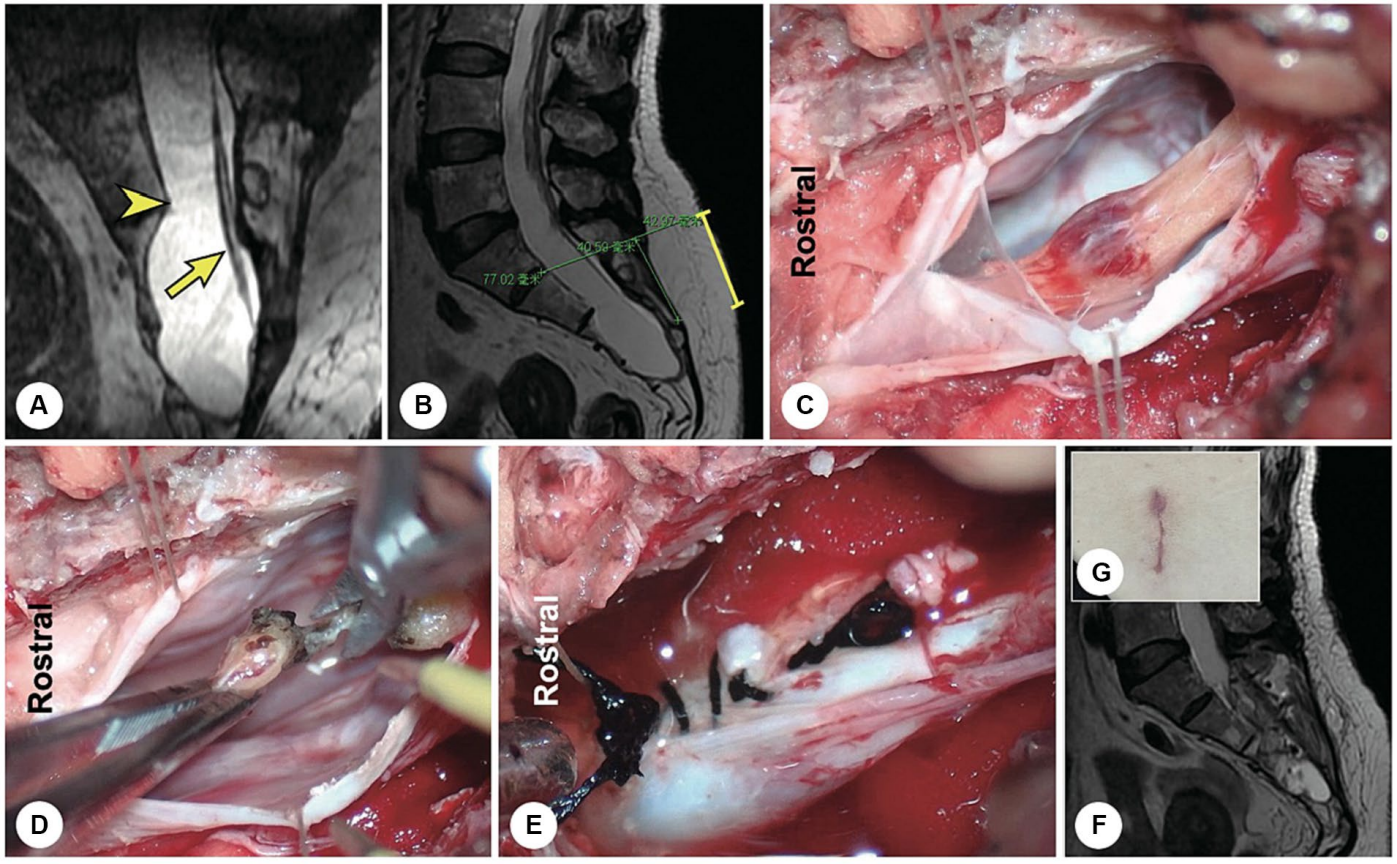
A representative patient with a filum terminale cyst. **(A)** Preoperatively, reconstructed spinal MR image showing a sacral cyst at the S2-4 level with a thickened filum terminale and wide leakage neck (the arrowhead indicates the cystic leakage orificium, and the arrow indicates the filum terminale). **(B)** A 4 cm precise minimally invasive incision was made (yellow line segment). **(C)** Intraoperatively, the filum terminale was found within the cyst cavity. **(D)** The filum terminale was cut off after electric coagulation. **(E)** The leakage orificium was ligated. **(F)** Follow-up MRI showed that the cyst had disappeared and that there was no recurrence. **(G)** The 4 cm long skin incision healed well.

**Figure 3:**
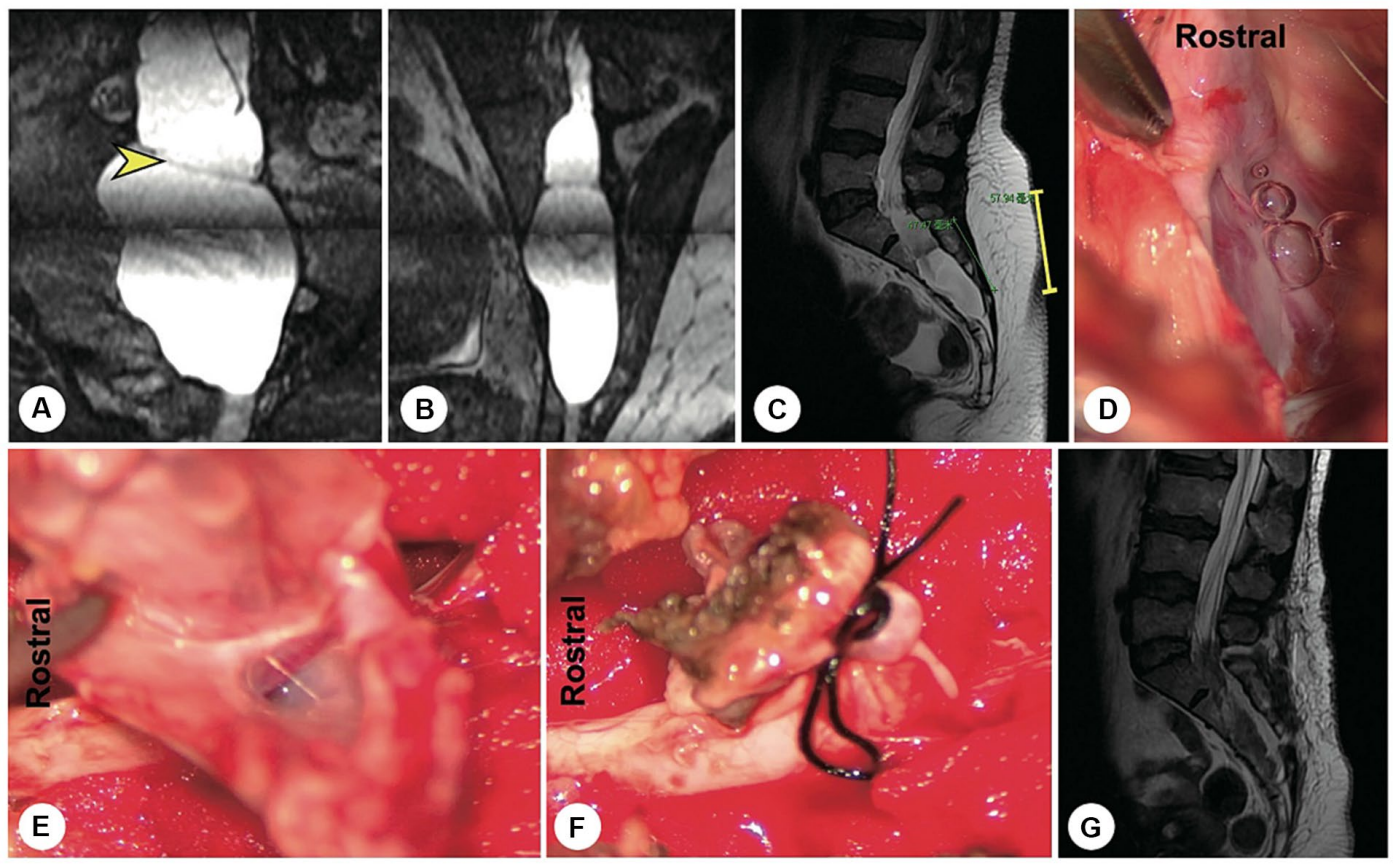
A representative patient with a fistula-type cyst. **(A,B)** Preoperatively, reconstructed MR image showing a fistula between the dural sac and the cyst (yellow arrowhead). **(C)** A 5 cm precise minimally invasive incision was designed (yellow line segment). **(D)** Intraoperatively, a fistula-type cyst was found, and there was no nerve root. **(E)** The cyst wall was dissected and turned over. **(F)** The cyst neck was ligated. **(G)** Follow-up MRI showed that the cyst had disappeared and that there was no recurrence.

**Figure 4:**
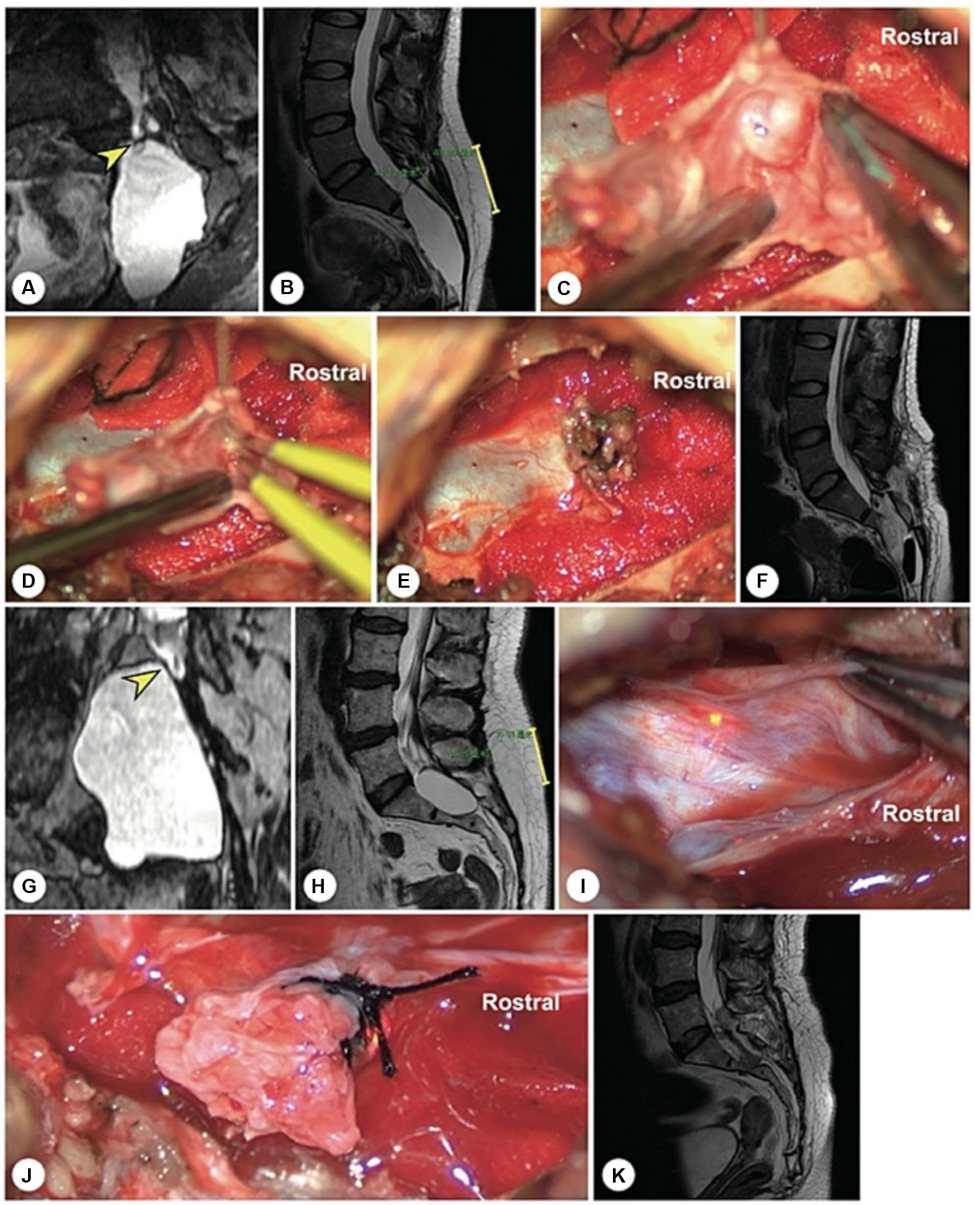
A representative case of an arachnoid hernia-type cyst and a representative case of a spinal dura mater leakage cyst. **(A)** Preoperatively, reconstructed MR image revealing an arachnoidal structure within the cyst (the yellow arrowhead indicates the cystic leakage orificium). **(B)** A 4 cm precise and minimally invasive incision was made (yellow line segment). **(C–E)** Intraoperatively, an arachnoidal diverticulum was found as the origin of the cerebrospinal fluid, and this cystic leakage orificium was ligated. **(F)** Follow-up MRI showed that the cyst had disappeared and that there was no recurrence. **(G)** Preoperatively, reconstruction MRI showed that the narrow end of the dural sac was squeezed to the left by a giant cyst without nerve roots (the yellow arrowhead indicates the cystic leakage orificium). **(H)** A 4 cm precise minimally invasive incision was made (yellow line segment). **(I)** Intraoperatively, we found that the lateral wall of the dural sac was weak, leading to leakage. **(J)** The cyst wall was dissected and turned over. **(K)** Follow-up MRI showed that the cyst had disappeared and that there was no recurrence.

All 40 patients (100.0%) presented with chronic lumbosacral or perineal pain, and 22 (55.0%) patients presented with numbness in the perianal region and lower extremities. Preoperatively, 15 patients had no bowel or bladder dysfunction, and none of them experienced any functional deterioration after surgery. There were varying degrees of bowel and/or bladder dysfunction in 25 patients, among whom 15 (37.5%) had mild dysfunction, such as urinary frequency and constipation; 7 (17.5%) had moderate dysfunction, including difficulty urinating and defecating; and 3 (7.5%) had incontinence. These patients were discharged after removal of the urethral catheter on postoperative days 7–10, and routine nutritional and neural rehabilitation therapy was continued. During the follow-up period, 15 patients achieved completely normal bowel and bladder functions, and 10 patients experienced varying degrees of improvement (Figures 2–4).

### Surgical treatment

**Figure 5:**
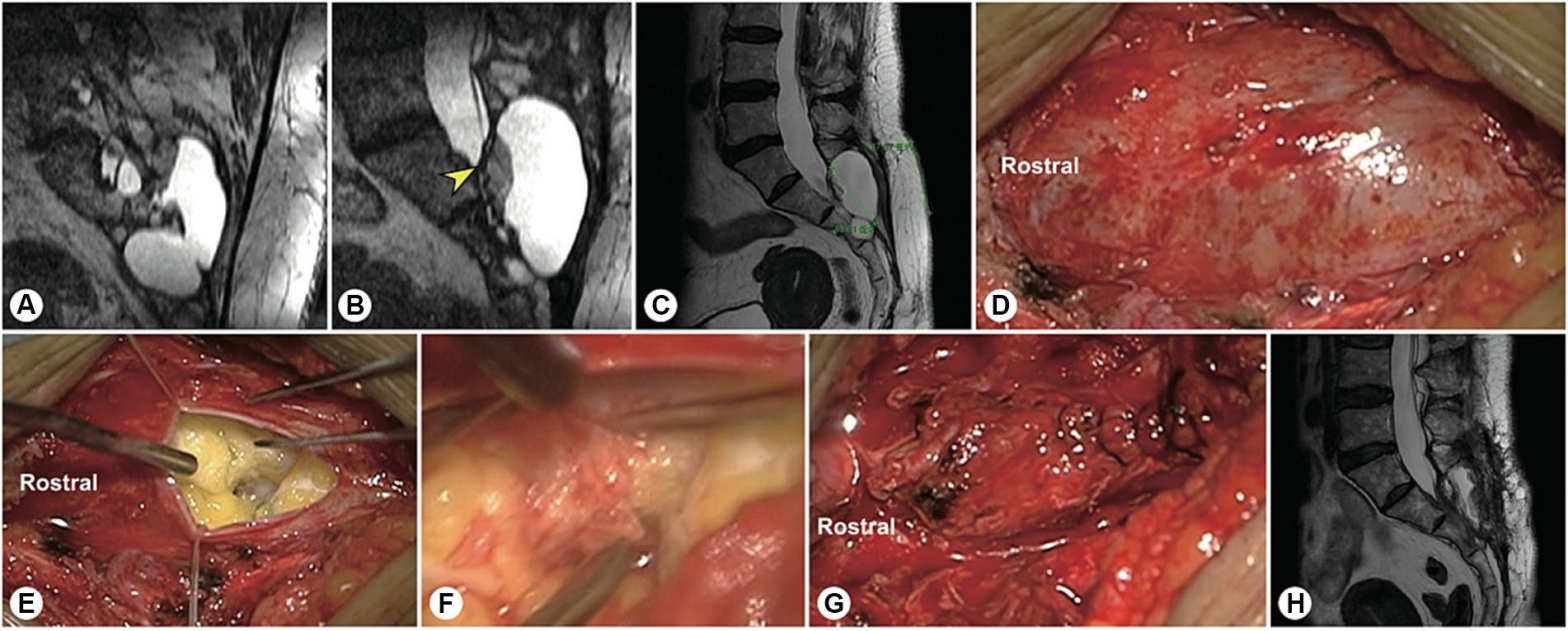
A representative patient with a massive sacral meningeal cyst. A 56 years-old woman with a massive sacral cyst was treated with glue injection and fat filling at the local hospital. **(A)** After referral to our institute, reconstructed MR image showed a sacral meningeal cyst and an extrasacral cyst, which were separated by the filled fat mass. **(B)** Sagittal spinal T2-weighted MR image showing the distorted dura sac end, dorsal fat mass, and residual cyst. **(C)** The precision of the incision is indicated by the green line. **(D)** Intraoperatively, a pseudocyst was found under the muscle layer. **(E)** After the pseudocyst was opened, cerebrospinal fluid was found to leak from the centre of the tightly packed fat. **(F)** After the filled fat was separated, the hardened biological glue was exposed. **(G)** After the neck of the cyst leakage site was sutured, the end of the dural sac was reinforced by an artificial dural membrane. **(H)** Two months after surgery, repeated MRI showed no cyst recurrence.

Four patients had received fat or muscle grafting and biological glue fixation at local hospitals, among whom one patient also underwent lumbar-sacral vertebral reinforcement internal fixation. However, postoperative recurrence of the cyst was noted, and the patient was referred to our institute. After comprehensively evaluating the cysts and involved nerve roots via reconstructed magnetic resonance imaging, precise minimally invasive surgery was performed. The leakage orificium was accurately identified and ligated. Postoperatively, the cysts had disappeared, and the symptoms were relieved (Figure 5). During the follow-up, no cyst recurrence was demonstrated.

### Representative complicated cases

**Figure 6:**
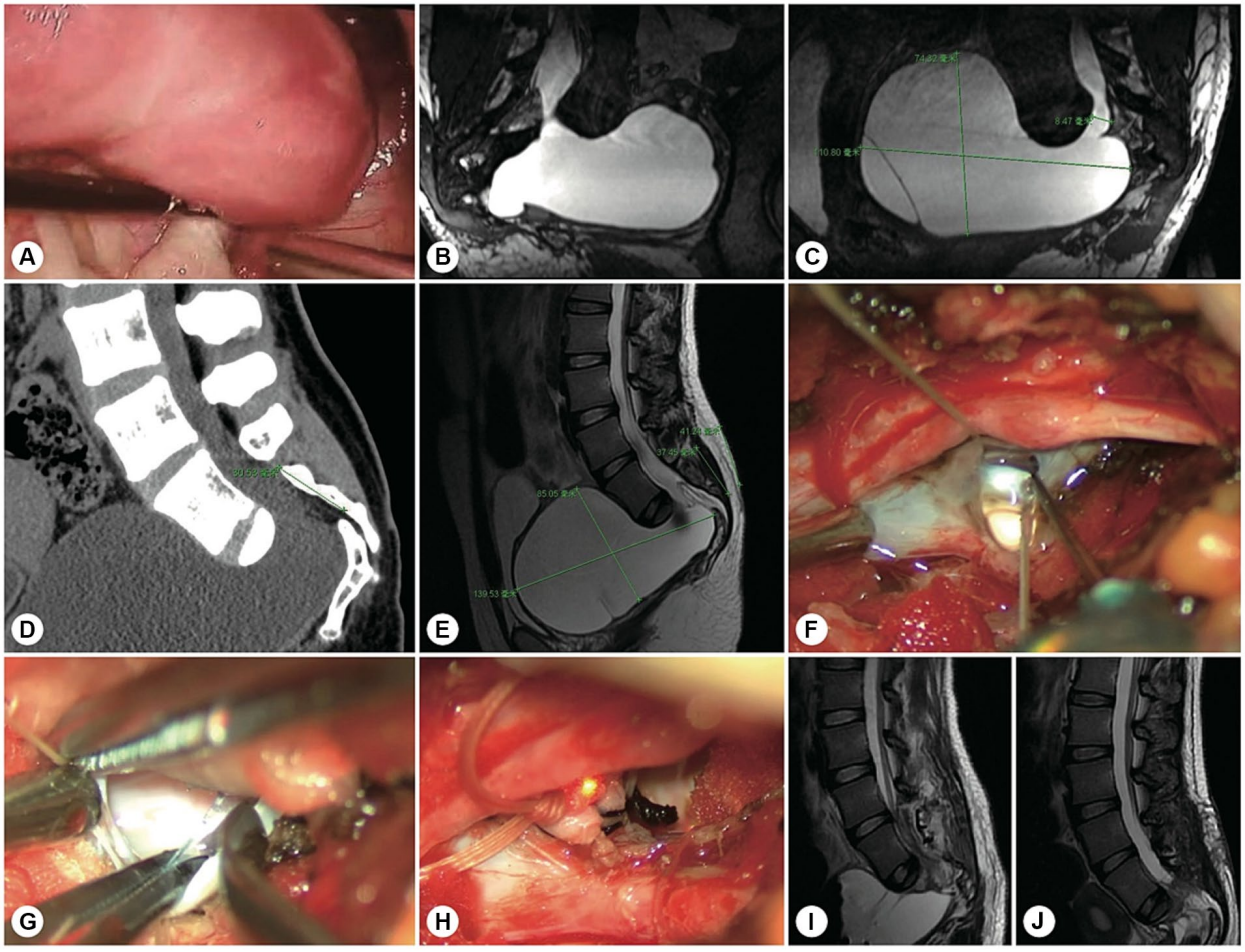
A representative patient with both sacroanterior and sacral cysts. A 22 years-old woman presenting with abdominal pain was found to have a pelvic mass. **(A)** Laparoscopic exploration revealed that the pelvic lesion was connected to the sacral canal. **(B)** Coronal MR image showing wide neck leakage between the end of the dural sac and the sacral canal. **(C)** Sagittal MR image revealing a giant sacroanterior cyst connected to a sacral cyst without nerve root fibres inside. **(D)** Sagittal CT image showing a sacroanterior bone defect. **(E)** A precise incision was made. **(F)** Intraoperatively, abundant cerebrospinal fluid leaked from the orificium, suggesting high intracystic pressure. **(G)** No spinal nerve root fibres were identified in the leakage orificium. **(H)** The leakage orificium was ligated. **(I)** On MRI, 1 week after the operation, the sacroanterior cyst had shrunk. **(J)** Three months after surgery, the sacroanterior cyst had almost completely disappeared on MRI.

Two female patients with sacroanterior cysts were misdiagnosed with pelvic masses during their first surgeries, both of whom underwent laparoscopic exploration at local hospitals. The first is a 22 years-old woman who presented with sacrococcygeal pain and sphincter disturbances. MRI revealed a large sacroanterior cyst originating from the sacral canal (Figure 6). After referral to our hospital, high-resolution spherical arbitrary-dimensional reconstructed MRI demonstrated that the cyst leakage orificium originated from the end of the dura mater and that there were no SNRFs within the cyst. With a precisely designed skin incision and bone window, the leakage orificium was ligated. Six months after surgery, the sacroanterior and sacral cysts had disappeared completely.

**Figure 7:**
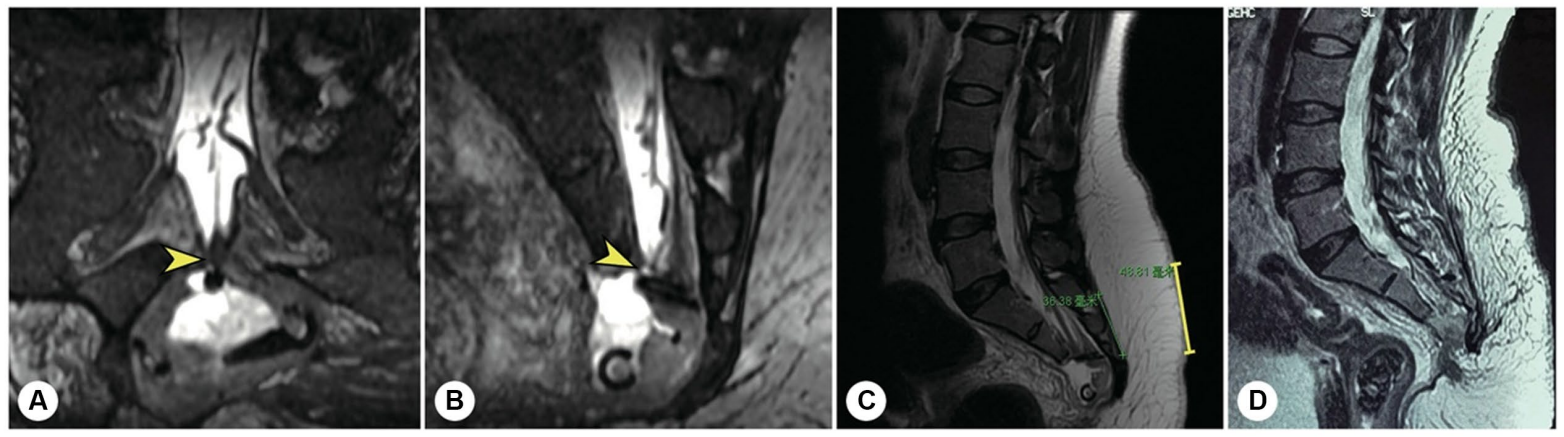
The other representative patient had both sacroanterior and sacral cysts. A 45 years-old female presenting with abdominal pain was found to have a pelvic mass on CT at the local hospital. The intrapelvic part of the cyst was resected under laparoscopy. **(A)** After referral to our institute, coronal reconstructed MR image showed a narrow-neck leakage orificium (yellow arrowhead) between the end of the dural sac and the sacral canal cyst, and the filum terminale was thickened. **(B)** Sagittal reconstructed MR image showing the leakage orificium (yellow arrowhead) at the end of the dural sac connected to the sacral cyst and the residual sac. **(C)** A 4 cm precise incision was made (yellow line segment). **(D)** After the leakage orificium was ligated and the tethered spinal cord was released, a follow-up MRI showed that the cyst had completely vanished.

The other patient was a 45 years-old female whose sacroanterior cyst in the pelvic cavity had been partially resected during the first exploratory surgery. Intraoperatively, the local surgeon attempted to clip the leakage orificium of the cyst with a titanium clip. Unfortunately, postoperative cerebrospinal fluid leakage occurred, and she was then transferred to our hospital. High-resolution spherical arbitrary-dimensional reconstructed MR images confirmed that the sacroanterior and sacral cysts originated from the end of the dura mater and contained no SNRFs. With a precise surgical incision along the midline of the sacrum, the cyst neck was accurately ligated. The patient recovered completely during the follow-up (Figure 7).

## Discussion

SMCs refer to diverticula of the arachnoid mater, dura mater, or nerve root sheath that may occur in the perineural, extradural, or intradural regions, respectively. These entities are relatively rare, accounting for approximately 1% of all spinal lesions (12). Previously, SMCs were also referred to as arachnoid cysts or meningeal diverticula. The majority of SMCs are asymptomatic and are detected incidentally via MRI. Michael Nabors classified spinal cysts into three categories: (a) meningeal cysts, (b) nonmeningeal epidural cysts, and (c) neurenteric cysts. Spinal meningeal cysts are further divided into three subtypes: (a) type I, extradural meningeal cysts containing no SNRFs, including extradural arachnoid cysts (type Ia) and sacral meningoceles (type Ib); (b) type II, extradural meningeal cysts containing SNRFs; and (c) type III, intradural meningeal cysts (13). In 2013, we summarized our previous clinical experience in the treatment of type I and type II SMCs (2, 3). In 2016, we proposed a distinct subtype of smooth muscle cells (SMCs), filum terminale cysts without SNRFs, which are usually accompanied by spinal cord tethering (4). For these patients, the surgical strategy should not be limited to suturing or ligating the cyst neck but should include releasing the tethered spinal cord.

The surgical goal for treating SMCs without SNRFs is to restore the normal anatomical structure of the terminal cisterna and the nerve root sleeve and to suture or ligate the neck or fistula of the cyst (14). Previously, several scholars have used procedures such as puncture, suction, and injection of glue to treat smooth muscle cells (SMCs) (9, 15, 16). These methods cannot reconstruct the anatomical structure of the nerve root sleeve, and blind puncture may stimulate or even injure the nerve root. In particular, these procedures are associated with a high recurrence rate, which seriously affects patients’ quality of life (8, 17, 18). In our cohort, four patients underwent reoperation after repeated aspiration, multiple injections of glue, or filling with fat and muscle in local hospitals. Additionally, filled biological glue and fat may cause space-occupying effects, compressing normal nerve structures and affecting the function of SNRFs (19). Moreover, these procedures may lead to local adhesions, inevitably aggravating symptoms after the operation (20).

Intraoperative suturing or ligation of the neck or fistula of the cyst is essential for SMCs without SNRFs. To prevent further impairment of the cyst wall by the SNRFs, the remaining cyst was left. The precise incision design is predicated on the accurate location of the cyst leakage orificium with the assistance of reconstructed MRI (21). In the present study, we divided the SMCs without SNRFs into four types: filum terminale cysts, accounting for 37.5%; arachnoid hernias, accounting for 35.0%; spinal dura mater leakage, accounting for 15.0%; and simple fistulas, accounting for 12.5%. The optimal surgical approach for various subtypes of patients should be distinct and individualized and should be adjusted appropriately. For filum terminale cysts, the internal or external filum terminale should be cut, while the cyst neck should be sutured or ligated, after which the filum terminale is released. For arachnoid hernia cysts, electrocautery and ligature were performed sequentially to reinforce the arachnoid membrane. For simple fistulas, direct suturing and ligatures were sufficient. For spinal dura mater leakage, after suturing and ligature, the dural sac end was reinforced (1, 22).

Some SMCs may be accompanied by sacroanterior cysts. The sacral and sacroanterior portions are usually connected, and the joint between the sacral canal and the dura mater is often the origin of the leakage orificium. As the intrasacral portion of the cyst gradually increases in size and breaks through the boundary of the sacroanterior bone, the sacroanterior region also becomes involved. Clinically, sacroanterior cysts often manifest as abdominal pain. Preoperative sacrococcygeal MRI is strongly recommended for diagnosis. For these patients, laparoscopic exploration and removal of the pelvic mass are highly risky and may cause irreversible and life-threatening continuous cerebrospinal fluid leakage and neurological infections (23). In our experience, complicated sacroanterior-sacral cysts without SNRFs can be effectively treated by suturing or ligating the leakage via a posterior approach. The cyst may disappear gradually after the operation. In the future, with the development of molecular biology techniques, additional disease-related biomarkers will be discovered, and nonsurgical treatment strategies may be developed (24, 25). However, currently, surgical treatment is the exclusive approach for relieving symptoms in a considerable proportion of patients (26, 27).

3D-FIESTA imaging is highly valuable for identifying nerve roots and leakage orificia (28). This sequence can compensate for the deficiencies of T1-FLAIR and T2WI, and the nerve roots often exhibit clear hypointensity, resulting in a sharp contrast with the hyperintensity of the cystic fluid. Moreover, this sequence is a 3D scan that can be reconstructed in any spherical dimension and displays the three-dimensional anatomical relationship between nerve roots and cysts from different angles. Additionally, high-resolution curve planar reformation can reveal the complete nerve pathway that enters the sacral cyst in a curved manner, as well as the possible presence of leakage (28).

Based on 3D thin-slice axial T2-weighted imaging, repeated spherical reconstruction in arbitrary dimensions can determine the number and trajectory of neural roots within the cyst as well as the location of the leakage orificium. The surgical incision and bone window were designed by setting the leakage orificium as the centre, and the length of the incision was adjusted according to the thickness of the soft tissue outside the posterior wall of the sacral canal. Then, with the use of an ultrasonic bone knife, the bone window was precisely opened, and after the cyst was processed, the posterior wall of the sacral canal was perfectly reset and fixed.

## Conclusion

Our study demonstrated the potential of using ANRR-MRI for accurate diagnosis and treatment of SMCs without SNRFs. With the use of this technique, we were able to accurately predict the location and size of the leakage orificium preoperatively and formulate precise surgical plans. However, additional studies with larger sample sizes are needed to confirm the effectiveness of this approach and to explore its potential in other fields of neurosurgery.

## Data availability statement

The original contributions presented in the study are included in the article/supplementary material, further inquiries can be directed to the corresponding author.

## Ethics statement

The studies involving humans were approved by Institutional Review Board and Ethics Committee of Peking University Third Hospital. The studies were conducted in accordance with the local legislation and institutional requirements. The participants provided their written informed consent to participate in this study.

## Author contributions

CY: Conceptualization, Data curation, Funding acquisition, Investigation, Methodology, Writing – original draft, Writing – review & editing. XL: Data curation, Investigation, Methodology, Writing – original draft, Writing – review & editing. LH: Data curation, Formal analysis, Investigation, Methodology, Writing – review & editing. QM: Data curation, Formal analysis, Investigation, Methodology, Writing – review & editing. XY: Data curation, Formal analysis, Investigation, Methodology, Writing – review & editing. QZ: Data curation, Formal analysis, Investigation, Methodology, Writing – review & editing. CW: Data curation, Formal analysis, Investigation, Writing – review & editing. HW: Writing – review & editing. JS: Conceptualization, Data curation, Formal analysis, Methodology, Supervision, Validation, Writing – original draft, Writing – review & editing.
